# Spilanthol from *Acmella Oleracea* Lowers the Intracellular Levels of cAMP Impairing NKCC2 Phosphorylation and Water Channel AQP2 Membrane Expression in Mouse Kidney

**DOI:** 10.1371/journal.pone.0156021

**Published:** 2016-05-23

**Authors:** Andrea Gerbino, Giorgia Schena, Serena Milano, Luigi Milella, Alan Franco Barbosa, Francesca Armentano, Giuseppe Procino, Maria Svelto, Monica Carmosino

**Affiliations:** 1 Department of Biosciences, Biotechnologies and Biopharmaceutics, University of Bari "Aldo Moro", 70126, Bari, Italy; 2 Department of Sciences, University of Basilicata, Viale dell'Ateneo Lucano 10, 85100, Potenza, Italy; 3 Department of Food Technology, Federal Rural University of Rio de Janeiro, BR 465 Km 07, Seropédica, Rio de Janeiro, Brazil; University of Geneva, SWITZERLAND

## Abstract

*Acmella oleracea* is well recognized in Brazilian traditional medicine as diuretic, although few scientific data have been published to support this effect. Aim of this study was to determine the molecular effect of *Acmella oleracea* extract and its main alkylamide spilanthol on two major processes involved in the urine concentrating mechanism: Na-K-2Cl symporter (NKCC2) activity in the thick ascending limb and water channel aquaporin 2 accumulation at the apical plasma membrane of collecting duct cells. Phosphorylation of NKCC2 was evaluated as index of its activation by Western blotting. Rate of aquaporin 2 apical expression was analyzed by confocal laser microscopy. Spilanthol-induced intracellular signalling events were dissected by video-imaging experiments. Exposure to spilanthol reduced the basal phosphorylation level of NKCC2 both in freshly isolated mouse kidney slices and in NKCC2-expresing HEK293 cells. In addition, exposure to spilanthol strongly reduced both desmopressin and low Cl^−^-dependent increase in NKCC2 phosphorylation in mouse kidney slices and NKCC2-expressing HEK293 cells, respectively. Similarly, spilanthol reduced both desmopressin- and forskolin-stimulated aquaporin 2 accumulation at the apical plasma membrane of collecting duct in mouse kidney slice and MCD4 cells, respectively. Of note, when orally administered, spilanthol induced a significant increase in both urine output and salt urinary excretion associated with a markedly reduced urine osmolality compared with control mice. Finally, at cellular level, spilanthol rapidly reduced or reversed basal and agonist-increased cAMP levels through a mechanism involving increases in intracellular [Ca^2+^]. In conclusion, **s**pilanthol-induced inhibition of cAMP production negatively modulates urine-concentrating mechanisms thus holding great promise for its use as diuretic.

## Introduction

Na^+^-K^+^-2Cl^−^-cotransporter (NKCC2) is responsible for 25% of the active sodium reabsorption in the kidney. It is, therefore, an important factor in the regulation of the circulating fluid volume and in long-term blood pressure control. The physiological importance of NKCC2 in the regulation of blood pressure has been well established with the use of loop diuretics such as bumetanide and furosemide that act as functional blockers of the cotransporter and are among the most powerful antihypertensive drug available to date [1].

However, their efficacy may decrease with time, and the chronic use of loop diuretics leads to activation of the renin-angiotensin system, which might worsen intra-renal hemodynamics [2].

For this reason new synthetic, semi-synthetic or natural sources (herbs and botanicals) of loop diuretics might be useful. In line with this there are an increasing number of published articles claiming that plants or plant-derived actives may function as mild diuretic agents [3, 4]. A large majority of this research has determined the degree of clinical support for the traditional use of common or folklore medicines. *Acmella oleracea*, known as jambu, was originally introduced from Brazil and nowadays cultivated and used medicinally in many parts of the world [5].

Extensive phytochemical investigations of *Acmella oleracea* had previously been reported. It constitutes a diverse group of compounds. Major isolates were lipophilic alkylamides or alkamides bearing different numbers of unsaturated hydrocarbons (alkenes and alkynes), such as spilanthol [6] also knonw as affinin (2E,6Z,8E)-N-isobutyl-2,6,8-decatrienamide [7]. Spilanthol is the main constituent isolated from many parts of *Acmella oleracea* [8] and it has been demonstrated to exert different biological activities e.g. antinflammatory [9]; antinociceptive without causing adverse effects [10] or penetration enhancing effect on model drugs [11]. On the other hand few papers suggested that the extracts obtained from different parts of *Acmella oleracea* could be useful for treating hypertension, in fact they demonstrated the vasorelaxant [12] and diuretic effects [13]. Ratnasooriya et al. demonstrated that the strong diuretic effect evoked by *Acmella oleracea* extract after 1 h was similar to that of furosemide and was accompanied by marked increases in both urinary Na^+^ and K^+^ levels. On one hand, these features strongly suggested that the *Acmella oleracea* extract could act as a loop diuretic reducing NKCC2 activity.

The release of the antidiuretic hormone arginine vasopressin (AVP) from the pituitary gland into the bloodstream elicits an antidiuretic action upon activation of the type-2 vasopressin receptor (AVPR2) [14], a G protein-coupled receptor expressed at the basolateral plasma membrane of the epithelial cells lining the Thick Ascending Limb of Henle (TAL), distal convolute tubules (DCT) and collecting ducts (CD). Once activated AVPR2 interacts with Gαs increasing intracellular cAMP levels and triggering a cascade of intracellular signals mostly mediated by PKA activation. In particular, AVP stimulates NaCl reabsorption in the TAL mainly through NKCC2 phosphorylation [15] whose increased activity contributes to the generation/maintenance of the cortico-medullary osmotic gradient thus providing the driving force for water reabsorption in kidney tubules. In addition, AVP elicits apical membrane exposure of the water channel aquaporin 2 (AQP2) [16] from a pool of intracellular storage vesicles in principal cells of the CD, significantly increasing water permeability at this site (for a review see [17]). Therefore, aim of this study was to determine the molecular effect of *Acmella oleracea* extract and its main alkylamide spilanthol on these two major processes involved in the urine concentrating mechanism: NKCC2 activity in the TAL and AQP2 accumulation at the apical plasma membrane of CD cells. Here we show for the first time that exposure to spilanthol reduces both NKCC2 phoshorylation and AQP2 plasma membrane accumulation by a Ca^2+^/cAMP interplay mechanism, leading to increased urine output and urinary salt excretion when orally administered.

## Materials and Methods

### Experimental animals

All animal experiments were performed in accordance with the Italian Institute of Health Guide for the Care and Use of Laboratory Animals, which conforms with the European Union Directive for the protection of experimental animals (2010/63/EU), and received approval from the Animal Experimentation Ethics Committee (CESA) of University of Bari "Aldo Moro", Italy. C57BL6/J mice were maintained on a 12 h light/12 h dark cycle, with free access to water and pelleted food.

### Evaluation of the diuretic activity

C57BL6/J mice were used to evaluate the effect of spilanthol on urinary parameters. Mice (n = 6 for each group) were kept in metabolic cages to measure 24h diuresis, osmolality and urine electrolytes. Animals were treated with spilanthol 800 mg/kg in food or vehicle alone. Urine collected was measured at the end of 24 h after treatment and total volume, osmolality and Na^+^, K^+^ and Cl^−^ in the urine were determined. Urinary electrolytes were measured by ion selective electrode method. Unpaired data were assessed for statistical significance using the Student’s t test. Significance was accepted for p values < 0.05.

### Solutions and materials

Anti-phosphatase buffer for cell lysis contained 150 mM NaCl, 30 mM NaF, 5 mM EDTA, 15 mM Na_2_HPO_4_, 15 mM pyrophosphate and 20 mM HEPES (pH 7.2) with 1% Triton X-100, supplemented with phosphatase and protease inhibitors (1:50, Roche, Basel, Switzerland). The low Cl^−^ solution used to activate NKCC2 contained 1 mM NaCl, 1 mM MgCl_2_, 1 mM Na_2_SO_4_, 1 mM CaCl_2_, 15 mM Na-HEPES and 134 mM Na-gluconate, pH 7.4. The Ringer’s solution used to perfuse cells during imaging experiment contained 140 mM NaCl, 5 mM KCl, 1 mM MgCl_2_, 10 mM Hepes, 5 mM Glucose, 1.0 mM CaCl_2_, pH 7.4. Cells were stimulated with a variety of drugs as described in the results, including ATP, cyclopiazonic acid and BAPTA-AM. Unless otherwise stated, all chemicals were purchased from Sigma-Aldrich (St. Louis, USA).

### Plant material and extract preparation

*Acmella oleracea* was collected in Igarapé-Açu (Pará State—Brazil; coordinates: 01° 07 '33'' S and 47° 37' 27'' W). A voucher specimen (MG205534) was deposited at the Museu Paraense Emílio Goeldi (www.museu-goeldi.br/), Belem, Brazil. No permits were required for *Acmella oleracea* collection. The plant flowers, leaves and stems were dried and comminuted. Cold-drying process was carried out in the climatised room with air conditioning (Midea, model MS2E-18CR, Brazil) at 25°C and by using the de-humidifier (Arsec, model 160, Brazil), in a 4 m^2^ room and it remained closed during the drying procedure. The plant material was grounded and then subjected to exhaustive extraction process by maceration with methanol (MeOH) at room temperature. The solvent was distilled in a rotary evaporator at 40°C under reduced pressure. The residue obtained of the MeOH extract, as previously proposed by Mbeunkui et al. [18], it was solubilised in MeOH/H_2_O (8:2) and the solution subjected to successive extractions in separatory funnel with the solvents n-hexane and dichloromethane.

### Chemical Analysis

The material was analyzed by a gas chromatograph coupled to a mass spectrometer—GC/MS (model QP-2010 Plus, Shimadzu, Japan) and by 1H and 13C NMR spectra data (model advance III, Bruker, Billerica, MA, USA). Dichloromethane (99.9%, HPLC grade) was used as the solvent in GC/MS analysis and CDCl3 (99%) was used as the solvent in NMR analysis. The GC/MS was equipped with a Factor Four/VF-5 ms fused-silica capillary column (30 m x 0.25 mm x 0.25 μm film thickness), using helium as carrier gas at 1 ml/min. The initial oven temperature was 100°C, which after being held constant for 40 min was increased at a rate of 10°C/min to 290°C, with a final isotherm (300°C) for 20 min. The sample injection volume was 1 μl (1:50 split mode). The injector and detector temperatures were both 300°C. The mass spectra were obtained in a range of m/z 10–300, by the electron impact technique at 70 eV. The quantitative analysis of the samples’s chemical composition was carried out in a HP 5890 Series II gas chromatograph with flame ionization detector (FID), using the same operational conditions and the same type of column as in the GC/MS analysis, with exception of the injector and detector temperatures that were of 250 and 300°C, respectively. The percentage of each constituent was calculated by the integral area under the respective peaks in relation to the total area of all the sample constituents. The identification of the major constituent was done based on the information obtained from the mentioned analytic methods, together with proton NMR (500 MHz) and carbon-13 NMR (125 MHz) spectra data analysis, obtained in a Bruker spectrometer, and comparison with literature data.

### MTT Assay

To test the cytotoxic effects of both the methanol plant extract (MPE) and spilanthol, the cell viability was determined using the (3-[4,5-dimethylthiazol-2-yl]-2,5-diphenyltetrazolium] bromide) (MTT) as a substrate, a yellow, water-soluble tetrazoliumdye. MTT is taken up into cells and reduced, mainly by mitochondrial enzymes of living cells, to yield a purple water-insoluble formazan product. Briefly, HEK293 cells were seeded into a 96-well tissue culture plate at a density of 1 x 10^4^ cells/well at 37°, and exposed to varying concentrations of either MPE or spilanthol (10, 50, 100, 200, 300, 400 μg/ml) for 24 h. Cells incubated with vehicle alone were used as negative control. At the end of treatment, cells were washed twice with phosphate-buffered saline (PBS), replenished with 100 μl of media containing 10 μl of MTT (5 mg/ml) and incubated in the dark for 4 h at 37°. The resulted formazan crystals were dissolved in 100 μl of DMSO and the absorbance intensity was measured by MULTISKAN GO Microplate Reader (Thermo Fisher Scientific, Waltham, MA, USA) at 560 nm, with a reference wavelength of 750 nm. All experiments were performed in triplicate and the cell viability was expressed as a percentage relative to untreated control cells.

### Kidney slices: preparation and treatment

Briefly, adult C57BL/6J male mice were anesthetized with an intraperitoneal. injection of tribromoethanol (250mg/kg) and euthanized by cervical dislocation. Kidneys were quickly removed, and sections of approximately 250 μm were cut using a McILWAIN Tissue Chopper (Ted Pella, Inc., Redding, Ca, USA). Kidney slices were incubated at 37°C for 15 min in Dulbecco’s modified Eagle’s medium-GlutaMAX^™^ (Thermo Fisher Scientific, Waltham, MA, USA) containing 20 mM HEPES sodium salt. Medium was previously pre-equilibrated in a 5% CO_2_ incubator. After equilibration, slices were stimulated for 2h at 37°C either with spilanthol (100 μg/ml), MPE (100 μg/ml) or with the vehicle alone (DMSO), in the same medium. To mimic physiological conditions another set of slices was stimulated for 30 min with spilanthol (100 μg/ml) either alone or followed by a 40 min incubation with desmopressin (dDAVP, 100 nM). Control slices were either stimulated with dDAVP (100 nM) or with the vehicle alone. Treated slices were then divided into two groups, one subjected to western blotting and one to immunofluorescence analysis.

### Cell culture and treatment

HEK293 cells stably expressing NKCC2 (NKCC2-HEK293) [19] were maintained in Dulbecco’s modified Eagle’s medium high glucose, GlutaMAX^™^ (Thermo Fisher Scientific, Waltham, MA, USA), 10% fetal bovine serum, penicillin (50 U/ml) and streptomycin (50 U/ml) at 37°C, 5% CO_2_ in a humidified incubator. Cells were grown until ~80% confluence and then treated either with spilanthol or MPE (100 μg/ml in growth medium) or with the vehicle alone (DMSO), for 24h at 37°C, 5% CO_2_. Concentration of use was selected based on MTT measurements on cells viability. When required, NKCC2 activation was induced by a 1 h incubation at 37°C, 5% CO_2_ in low Cl^−^ medium either in combination with spilanthol (100 μg/ml in growth medium) or vehicle alone, as control condition. Mouse cortical collecting duct MCD4 cells stably expressing human AQP2 were generated and cultured as described elsewhere [20–22]. To evaluate the effect of spilanthol on AQP2 accumulation at the apical plasma membrane, MCD4 cells were incubated with 100 μM forskolin alone for 30 min or after 1h preincubation with spilanthol (100 μg/ml in growth medium) both at 37°C, 5% CO_2_. AQP2 localization was detected by immunofluorescence followed by confocal laser-scanning microscopy.

### Cell and tissue fractionation and immunoblotting

All samples, both from cells and tissues, were processed as follow: cells or slices were lysed in ice-cold antiphosphatase buffer and sonicated for 15 sec. Slices were lysed for 60 additional min on ice. Unsolubilized material was pelleted by centrifugation at 13,000 g for 30 min at 4°C. Supernatants were separated by standard SDS-PAGE and analyzed by western blotting. Samples were resolved on 8% homemade polyacrylamide gel. After blocking with 3% bovine serum albumin in TRIS buffer saline-tween 20 (TBS-T), blots were incubated overnight at 4°C with the following antibodies in blocking buffer: antibody *vs* p-NKCC2 (dil. 1:500) and *vs* total NKCC2 (dil.1:500, cat. #3562P, Millipore, Billerica, MA, USA). Membranes were washed and incubated with horseradish peroxidase–conjugated secondary antibody. Negative controls with secondary antibodies alone were performed (not shown). Antibody *vs* p-NKCC2 was kindly provided by Prof. Biff Forbush from the Department of Cellular and Molecular Physiology, Yale University, New Haven, CT, USA. Target proteins were revealed with an enhanced chemiluminescent detection system superSignal West Pico Chemiluminescent Substrate (Thermo Fisher Scientific, Waltham, MA, USA). Chemiluminescence was detected with Chemidoc XRS detection system imaged with Image Lab Software (Bio-Rad, Hercules, California, U.S.A.) and quantified with ImageJ software (http://imagej.nih.gov/ij/).

### Immunofluorescence confocal analysis

Mouse kidney slices were fixed by immersion in 4% paraformaldehyde in PBS at 4°C overnight, cryopreserved in 30% sucrose in PBS for 12 h and then embedded in optimal cutting temperature medium. Ultra-thin sections (10μm) collected on Superfrost/Plus Microscope Slides (Thermo Fisher Scientific, Waltham, MA, USA) were subjected to immunofluorescence analysis as follow: non-specific binding sites were blocked with 1% bovine serum albumin in PBS for 30 min at room temperature. Sections were then incubated with primary antibodies *vs* pNKCC2 (dil. 1:500), *vs* AQP2 (Eurogentec, Seraing, Liège, Belgium, dil: 1:1000) and *vs* Tamm-Horsfall Protein (Santa Cruz Biotechnology, Dallas, USA, dil: 1:100) overnightat 4°C in the same solution. The following day sections were washed in PBS and incubated with the appropriate 488 Alexa-Fluor-conjugated secondary antibody (Thermo Fisher Scientific, Waltham, MA, USA) for 1h at room temperature. Confocal images were obtained with a confocal laser-scanning fluorescence microscope Leica TSC-SP2 (Leica Microsystem, Wetzlar, Germany). NKCC2-HEK293 and MCD4 cells seeded on glass coverslips were fixed in ice-cold methanol for 5min, washed in PBS and subjected to immunofluorescence analysis (antibodies *vs* pNKCC2, dil. 1:500, and *vs* AQP2, dil. 1:3000) as described before. Rabbit affinity-purified polyclonal antibody against human AQP2 was previously described [23].

### FRET-based measurement of cAMP in single cells

Intracellular cAMP level was imaged in single cells using Epac H90 [CFP(nd)-EPAC(∂DEP/CD)-cp173Venus(d)] [24]. This reporter (kindly provided by Prof. Kees Jalink, Netherlands Cancer Institute) is a soluble monomeric construct that relies on conformation-dependent FRET between YFP- and CFP-labeled fragments of the Epac protein. NKCC2-HEK293 cells were transiently transfected with Epac H90 using the Lipofectamine 2000 transfection reagent (Thermo Fisher Scientific, Waltham, MA, USA). Real-time digital imaging measurements of the 480 nm-to-535 nm FRET emission ratio (reflecting the degree of Epac conformational changes and, thus, intracellular cAMP levels) were carried out using a Metafluor-based imaging set-up described in the following section for intracellular Ca^2+^ measurements. 480/535 nm FRET emission ratio was recorded and normalized to basal fluorescence ratio observed in the absence of stimulus (R/R0). At the end of each experiment using the cAMP probe, cells were stimulated with a supramaximal dose of forskolin (5–10 μM), a reliable activator of adenylate cyclase (AC). Since cells were occasionally fluorescent but nonetheless nonresponsive to agonists (possibly because of improper folding or targeting of the probe), only the cells that responded to forskolin with a large ratio increase were statistically averaged. Data from 5–8 cells were summarized in a single experiment, and at least four independent runs were conducted. Paired data were assessed whenever possible for statistical significance using the Student’s t test. Data are expressed as means ± SEM with n equal to the number of experiments. P <0.05 was considered statistically significant for cAMP FRET experiments.

Steady state FRET experiments were performed as previously described [25]. Briefly, NKCC2-HEK293 cells were grown onto Ø 20mm glass coverslips at 37°C and transfected with Epac H90 using Lipofectamine 2000. Cells were either left under basal condition or stimulated for 30 min with forskolin (100μM) or alternatively with spilanthol at 100 μg/ml (60 min) in regular medium at 37°C. 48 hours post-transfection cells were fixed with ice-cold methanol and mounted on glass slides. Steady state FRET measurements were carried out using MetaMorph software (Molecular Devices, MDS Analytical Technologies, Toronto, Canada). It is important to note that cAMP production decreases the netFRET signal calculated using the following equation. NetFRET signal = FRET signal—a x YFP signal—b x CFP signal where a and b are the ratio of the signal in FRET channel to the signal in YFP channel in the absence of donor and to the signal in CFP channel in the absence of acceptor respectively. Data from 15 different fields each one containing at least 3 cells were summarized for a single coverslip/treatment, and at least three independent coverslips were blind-analyzed.

Unpaired data were assessed for statistical significance using the Student’s t test. Data are expressed as means ± SEM with n equal to the total number of cells analyzed. P <0.05 was considered statistically significant for cAMP FRET experiments.

### Intracellular Ca^2+^ measurements

For intracellular Ca^2+^ measurements, cells were seeded on Ø 40 mm glass coverslips. Either NKCC2-HEK293 or MCD4 cells were loaded with 5–7 μM Fura-2AM for 30 min at 37°C in DMEM. For fluorescence measurements, the coverslips with dye-loaded cells were mounted in a perfusion chamber (FCS2 Closed Chamber System, BIOPTECHS, Butler, U.S.A.) and measurements were performed using an inverted microscope (Nikon Eclipse TE2000-S microscope, Shinagawa, Tokyo, Japan) equipped for single cell fluorescence measurements and imaging analysis. The sample was illuminated through a 40X oil immersion objective (NA = 1.30). The Fura-2AM loaded sample was excited at 340 and 380 nm every 5 seconds. Emitted fluorescence was passed through a dichroic mirror, filtered at 510 nm (Omega Optical, Brattleboro, VT, USA) and captured by a cooled CCD camera (CoolSNAP HQ, Photometrics, Tucson, AZ, USA). Fluorescence measurements were carried out using Metafluor software (Molecular Devices, MDS Analytical Technologies, Toronto, Canada). Results are presented as the ratio of the fluorescence signal obtained upon excitation at 340/380 nm normalized to the basal fluorescence ratio observed in the absence of stimulus (R/R0). The bar graphs show the averaged rate of fluorescence ratio changes with respect to those elicited by ATP used in each experiment as internal control. Data from 20 to 40 cells were summarized in a single run, and at least three independent experiments were conducted.

Paired data were assessed for statistical significance using the Student’s t test. Data are expressed as means ± SEM with n equal to the number of experiments. P <0.05 was considered statistically significant for Fura-2 ratio imaging experiments.

## Results

### Isolation of spilathol from *Acmella oleracea*

To isolate spilathol, the main component of *Acmella oleracea*, MeOH plant extract (MPE) was subjected to successive extractions with n-hexane and dichloromethaneas solvents to obtain different plant fractions. All fractions were then subjected to detailed chemical analysis (see Methods) to identify their components.

**Fig 1:**
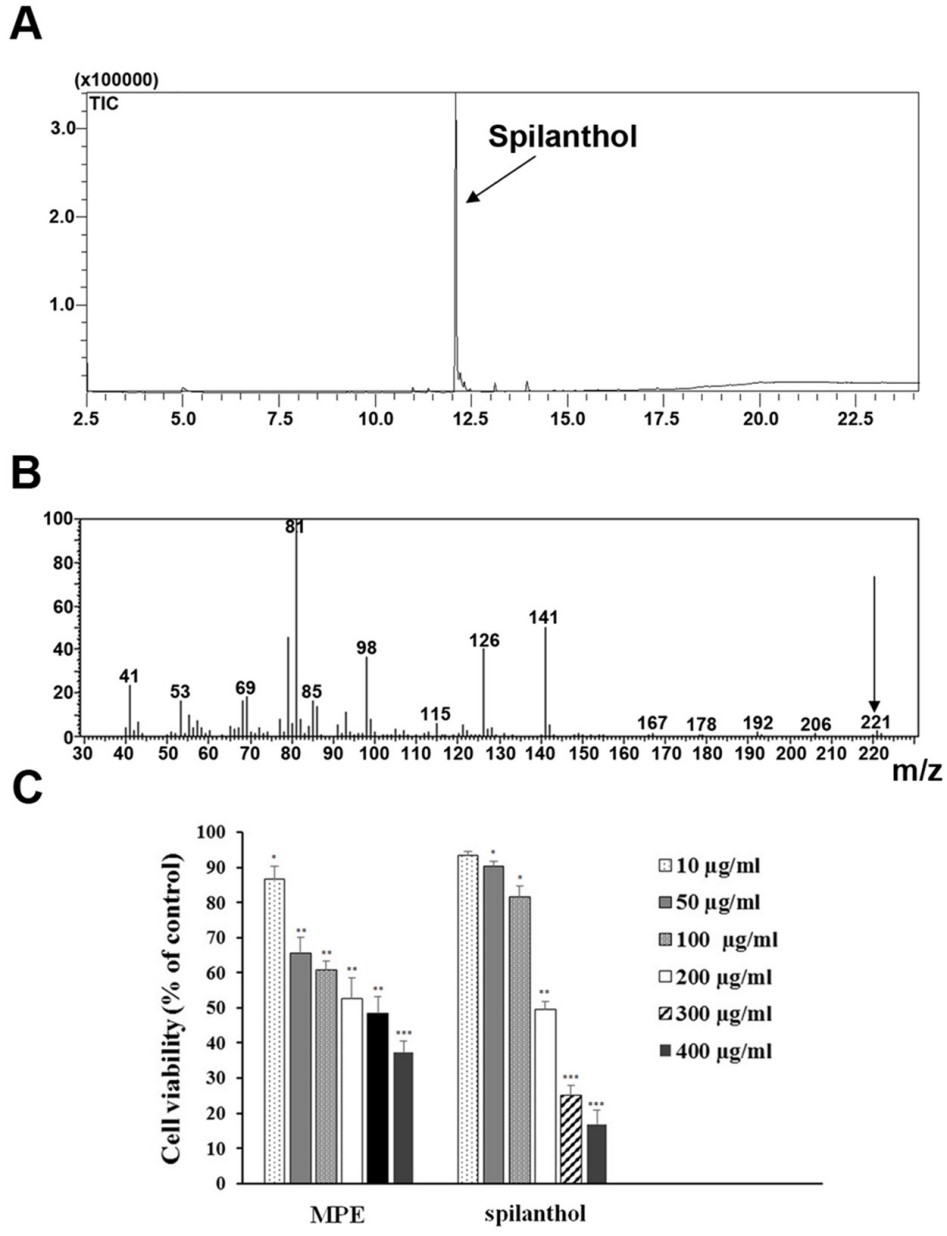
Isolation of spilanthol and evaluation of its cytotoxicity. **(A)** Chromatogram of sample containing spilanthol—dichloromethane fractions. **(B)** Mass spectrum of spilanthol. **(C)** Viability of HEK293 cells cultured in the absence and presence of diverse doses of either MPE or spilanthol. Cells were subjected to MTT assay as described in Materials and Methods. The cell viability of control cells was defined as 100% (not shown in the histogram). The reported values are mean ± SD from three independent experiments (*p<0.05, **p<0.01, ***p<0.001 *vs* control).

We found that Fraction 2 (F2) contains spilanthol with a 99% grade of purity. The percentage of the F2 main constituent was calculated by the integral area under the respective peaks in relation to the total area of all the sample constituents; the grade of purity of the main compound was 99% (Fig 1A). F2 was then subjected to MS analysis to identify the structure of the main compound. The electron impact ionization analysis MS spectra (Fig 1B) evidenced a molecular ion peak at m/z 221.04, corresponding to a molecular formula of C_14_H_23_NO, and two peaks at m/z 81.04 and 141.01 respectively, that are characteristic fragments coming from the spilanthol mass spectra. In fact, the two main fragments were generated from the amidic bond fragmentation that is normally observed in this kind of compounds [26].

As described above, the confirmation that F2 was spilanthol was also performed with proton NMR (500 MHz) and carbon-13 NMR (125 MHz) spectra data analysis, comparing our results with literature data (data not shown) [27]. Due to these results it is possible to confirm spilanthol as the main constituent of the dichloromethane fraction/F2 with 99% grade of purity.

### Citotoxicity assay

To identify the non-toxic concentration of both MPE and spilanthol, we performed the cytotoxic assay on HEK293 cells. As shown in Fig 1C, both MPE and spilanthol induced cell death in a dose-dependent manner as compared with vehicle controls. The IC50 values at 24 h for MPE and spilantol were 234 μg/ml and 260 μg/ml, respectively. Based on these results, in the following functional studies, we used both MPE and spilanthol at 100 μg/ml or lower, far below the IC50 values.

### Spilanthol inhibits NKCC2 phosphorylation under basal and stimulated conditions

**Fig 2:**
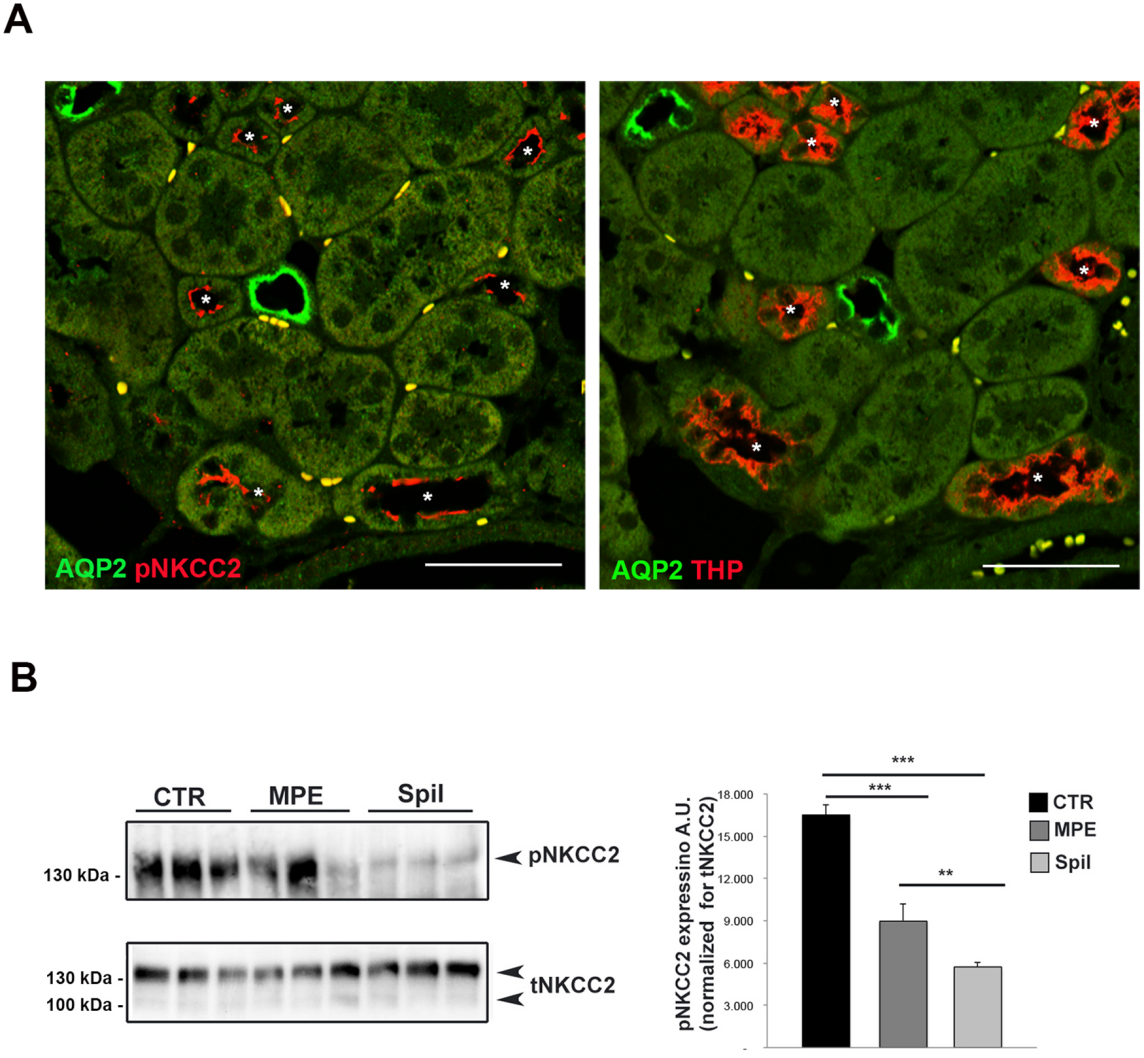
Exposure to spilanthol reduced basal NKCC2 phosphorylation (pNKCC2) in mouse kidney slices. **(A)** Coimmunostaining of R5 antibody (pNKCC2) with AQP2 as marker of collecting ducts (AQP2) and Tamm-Horsfall Protein as marker of TAL tubules (THP) in serial kidney sections. Asterisks indicate corresponding TAL tubules in the serial sections, Bar = 30 μm. **(B)** Kidney slices (250 μm) were stimulated 2h with either 100 μg/ml MPE or 100 μg/ml spilanthol, then lysed and total protein extracts analyzed for pNKCC2 expression as shown by this representative Western blot. **Right panel.** Densitometric analysis showed a significant reduction of pNKCC2 (normalized to total NKCC2) in kidney slices stimulated with either MPE (***p<0.001) or spilanthol (***p<0.001) compared to control condition. Spilanthol-induced reduction of pNKCC2 was significantly larger than that evoked by MPE (**p<0.01). Comparable results were obtained in two different mice preparations and significance calculated by Student**’**s T-test for unpaired data.

At first, we investigated the putative effect induced by spilanthol on NKCC2 whose activity, crucial for NaCl reabsorption in the TAL, drives the urine concentrating mechanism. We aimed at quantifying the phosphorylation of NKCC2 (pNKCC2) as index of its activation using an antibody that specifically recognizes the regulatory phospho-threonines 96 and 101 [28] (known as R5), required for NKCC2 activity [29]. Here, immunofluorescence experiments on freshly isolated kidney slice showed the expression and the proper apical localization of pNKCC2 in the TAL cells under unstimulated conditions (Fig 2A). R5 antibody signal was clearly confined at the apical membrane of AQP2-negative tubules (Fig 2A left panel) and in Tamm-Horsfall positive tubules visualized in the sequential slice (Fig 2A, right panel).

A semi-quantitative analysis of pNKCC2 on kidney slices lysates exposed to either 100 μg/ml MPE or 100 μg/ml spilanthol showed a significant reduction of pNKCC2 level compared to control conditions, as measured by Western blotting (Fig 2B, MPE, Spil).

Concentration below 100 μg/ml of both spilanthol and MPE resulted ineffective in modulating the NKCC2 phosphorylation in this experimental condition (data not shown).

Densitometric analysis showed a significant reduction of pNKCC2 (normalized to total NKCC2) in kidney slices exposed to either MPE (pNKCC2 expression: 8966.35 a.u. ± 1208.12 *vs* 16508.84 a.u. ± 726.45, ***p<0.001) or spilanthol (pNKCC2 expression: 5725.34 a.u. ± 327.14 *vs* 16508.84 a.u. ± 726.45, *** p<0.001) compared to control condition. Interestingly, the effect induced by spilanthol was significantly larger than that exerted by MPE (pNKCC2 expression: 5725.34 a.u. ± 327.14 *vs* 8966.35 a.u. ± 1208.12, ** p<0.01).

**Fig 3:**
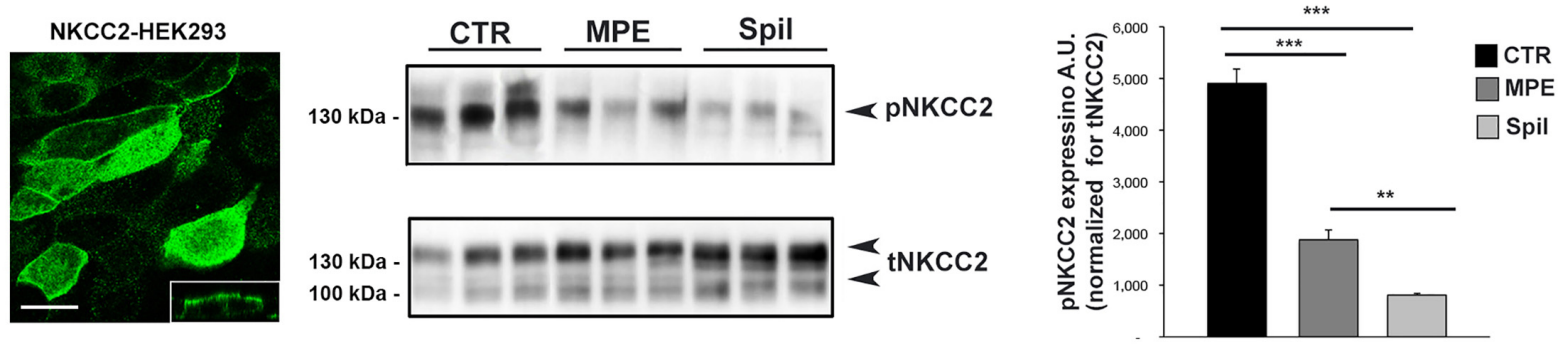
Exposure to spilanthol reduced basal NKCC2 phosphorylation (pNKCC2) in NKCC2 expressing HEK293 cells. **Left panel**. HEK293 cells stably transfected with NKCC2 were stained for NKCC2 and analyzed with confocal laser-scanning microscopy. Note the proper localization of NKCC2 in this cell line. Bar = 10 μm. **Middle panel.** NKCC2-HEK293 cells were stimulated overnight with either 100 μg/ml MPE or 100 μg/ml spilanthol, then lysed and total protein extracts analyzed for pNKCC2 expression as shown by this representative Western blot. **Right panel.** Densitometric analysis showed a significant reduction of pNKCC2 (normalized to total NKCC2) in NKCC2-HEK293 cells stimulated with either MPE (***p<0.001) or spilanthol (***p<0.001) compared to control condition. Spilanthol-induced reduction of pNKCC2 was significantly larger than that evoked by MPE (** p<0.01). Comparable results were obtained in three different experiments and significance calculated by Student**’**s T-test for unpaired data.

The same results were obtained in parallel experiments performed using HEK293 cells stably transfected with the full length NKCC2 (Fig 3, NKCC2-HEK293 cells).

Densitometric analysis showed a significant reduction of pNKCC2 (normalized to total NKCC2) in NKCC2-HEK293 cells exposed to either MPE (pNKCC2 expression: 1.88 a.u. ± 0.20 *vs* 4.90 a.u. ± 0.28, ***p<0.001) or spilanthol (pNKCC2 expression: 0.81 a.u. ± 0.04 *vs* 4.90 a.u. ± 0.28, ***p<0.001) compared to control condition (Fig 3, MPE, Spil). Spilanthol-induced reduction of pNKCC2 was significantly larger than that evoked by MPE (pNKCC2 expression: 0.81a.u. ± 0.04 *vs* 1.88a.u. ± 0.20, **p<0.01).

Control experiments in untransfected HEK293 cells were reported in S1 Fig.

Results obtained so far suggested that spilanthol is the main bioactive component of the plant extract able to impinge NKCC2 phosphorylation. We indeed, performed the following experiments using spilanthol.

Moreover, in NKCC2-HEK293 cells, spilanthol resulted effective also at lower doses as shown in the dose-response assay shown in the S2 Fig.

**Fig 4:**
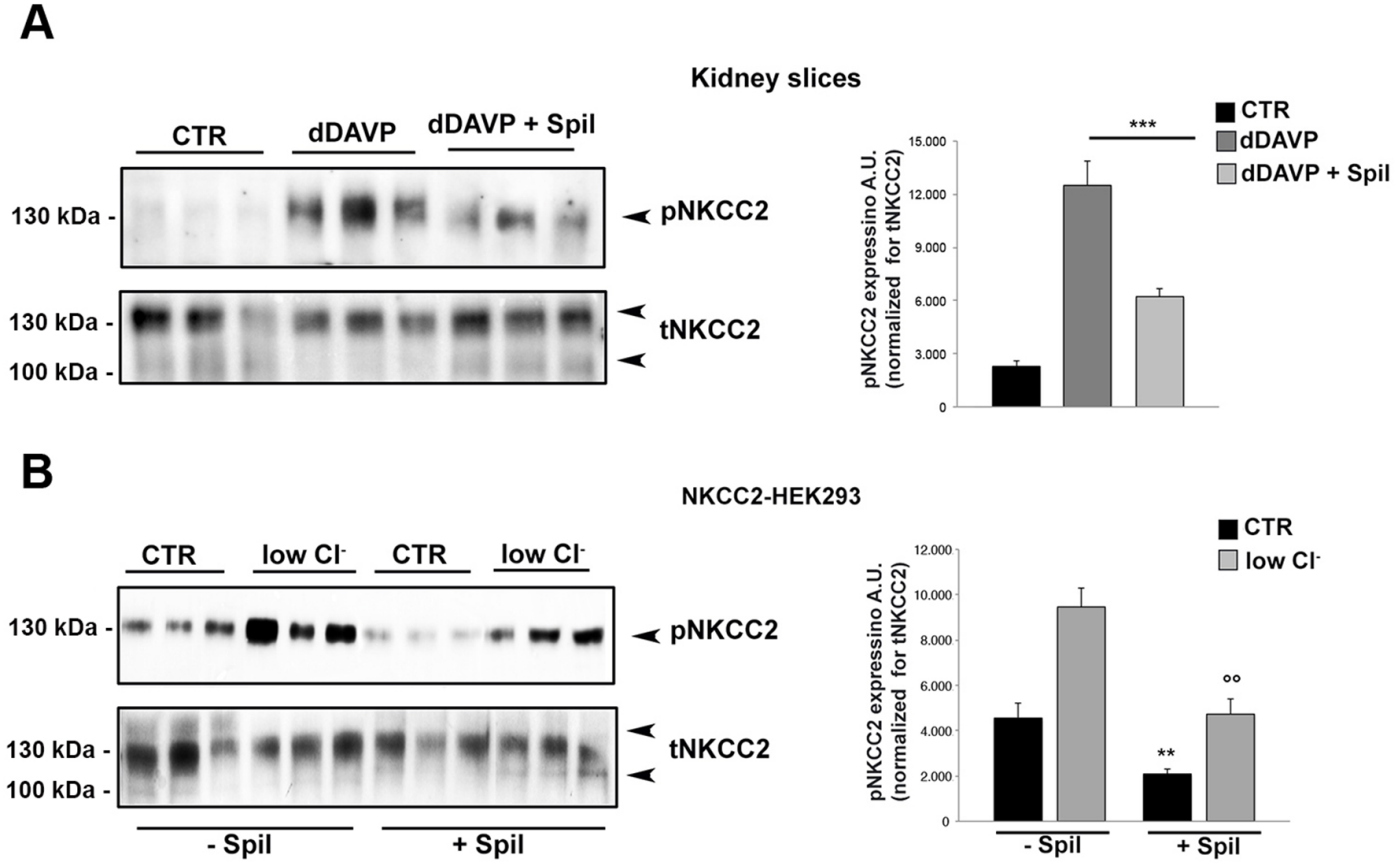
Exposure to spilanthol reduced both dDAVP- and low Cl^−^ stimulated NKCC2 activation in mouse kidney slices and NKCC2-HEK293 cells. **(A) Left panel.** Freshly-isolated kidney slices were stimulated 40 min with desmopressin (dDAVP) either in the absence (dDAVP) or in the presence of 100 μg/ml spilanthol (dDAVP + spil), then lysed and total protein extracts analyzed for pNKCC2 expression as shown by the representative Western blot. **Right panel.** The densitometric analysis normalized *vs* total NKCC2 showed that the significant increase of pNKCC2 induced by dDAVP (***p<0.001) was inhibited after pretreatment with spilanthol (***p<0.001) in kidney slices. Comparable results were obtained in two different mice preparation and significance calculated by Student**’**s T-test for unpaired data. **(B) Left panel.** NKCC2-HEK293 cells were stimulated with 1h incubation in low Cl^−^ medium either in the presence of sphilantol (100 μg/ml in growth medium) or not, as control condition. Cells were then lysed and total protein extracts analyzed for pNKCC2 expression as shown by this representative Western blot. **Right panel.** The densitometric analysis normalized *vs* total NKCC2 showed a significant increase of pNKCC2 in low Cl^−^-stimulated NKCC2-HEK293 cells when compared with unstimulated cells (***p<0.001). Pretreatment with 100 μg/ml spilanthol reduced the amount of pNKCC2 both under basal conditions (** p<0.01) and upon low Cl^−^ activation (°°p<0.01) relative to cells not exposed to spilanthol. Comparable results were obtained in three different experiments and significance calculated by Student**’**s T-test for unpaired data.

Next, we aimed at investigating whether spilanthol was also able to inhibit NKCC2 phosphorylation when the cotransporter was under intense activating conditions. It is known that desmopresin (dDAVP) enhances NKCC2 phosphorylation in the TAL primarily via AVPR2-mediated increase in intracellular cAMP [15]. We indeed, assayed the spilanthol effect on kidney slices treated with dDAVP by western blotting (Fig 4A). As expected the phosphorylation levels of NKCC2 in response to dDAVP significantly increased. However, when kidney slices were pretreated with 100 μg/ml spilanthol for 60 min, the dDAVP-induced increase of pNKCC2 recorded in stimulating conditions was significantly reduced (Fig 4A, dDAVP, dDAVP + Spil). The densitometric analysis of pNKCC2 normalized *vs* total NKCC2 showed that the significant increase of pNKCC2 induced by dDAVP (pNKCC2 expression: 2283.44 a.u. ± 313.53 *vs* 12505.68 a.u. ± 1397.62, *** p<0.001) was inhibited in kidney slices after pretreatment with spilanthol (pNKCC2 expression: 12505.68 a.u. ± 1397.62 *vs* 6221.56 a.u. ± 437.94, ***p<0.001).

Similar results were obtained in NKCC2-HEK293 cells where NKCC2 was maximally activated by incubating cells in a low Cl^−^ solution (Fig 4B). Intracellular Cl^−^ depletion activates NKCC2 by promoting the phosphorylation of three threonines (96, 101, and 111) in the amino terminus [30]. As expected the level of pNKCC2 clearly increased in response to low Cl^−^ stimulation (Fig 4B, low Cl^−^,—Spil) in NKCC2-HEK293 cells. On the other hand, in cells pretreated with 100 μg/ml spilanthol the amount of pNKCC2 was decreased both under basal conditions and upon low Cl^−^ activation relative to cells not exposed to spilanthol (Fig 4B, low Cl^−^, + Spil). The densitometric analysis of pNKCC2 normalized *vs* total NKCC2 showed a significant increase of pNKCC2 in low Cl^−^-stimulated NKCC2-HEK293 cells when compared with unstimulated cells (pNKCC2 expression: 4555.57 a.u. ± 667.12 *vs* 9457.29 a.u. ± 814.20, ***p<0.001). Pretreatment with 100 μg/ml spilanthol reduced the amount pNKCC2 both under basal conditions (pNKCC2 expression: 4555.57 a.u. ± 667.12 *vs* 2088.71 a.u. ± 200.00, **p<0.01) and upon low Cl^−^ activation (pNKCC2 expression: 9457.29 a.u. ± 814.20 *vs* 4728.98 a.u. ± 674.56,°°p<0.01) relative to cells not exposed to spilanthol.

Collectively these results suggest that: 1) *Acmella oleracea* extract may act as diuretic inhibiting the NKCC2 activity in the TAL; 2) spilanthol is the bioactive compound of *Acmella oleracea* responsible for its diuretic properties.

### Spilanthol reduces AQP2 apical expression in both MCD4 cells and kidney slices

**Fig 5:**
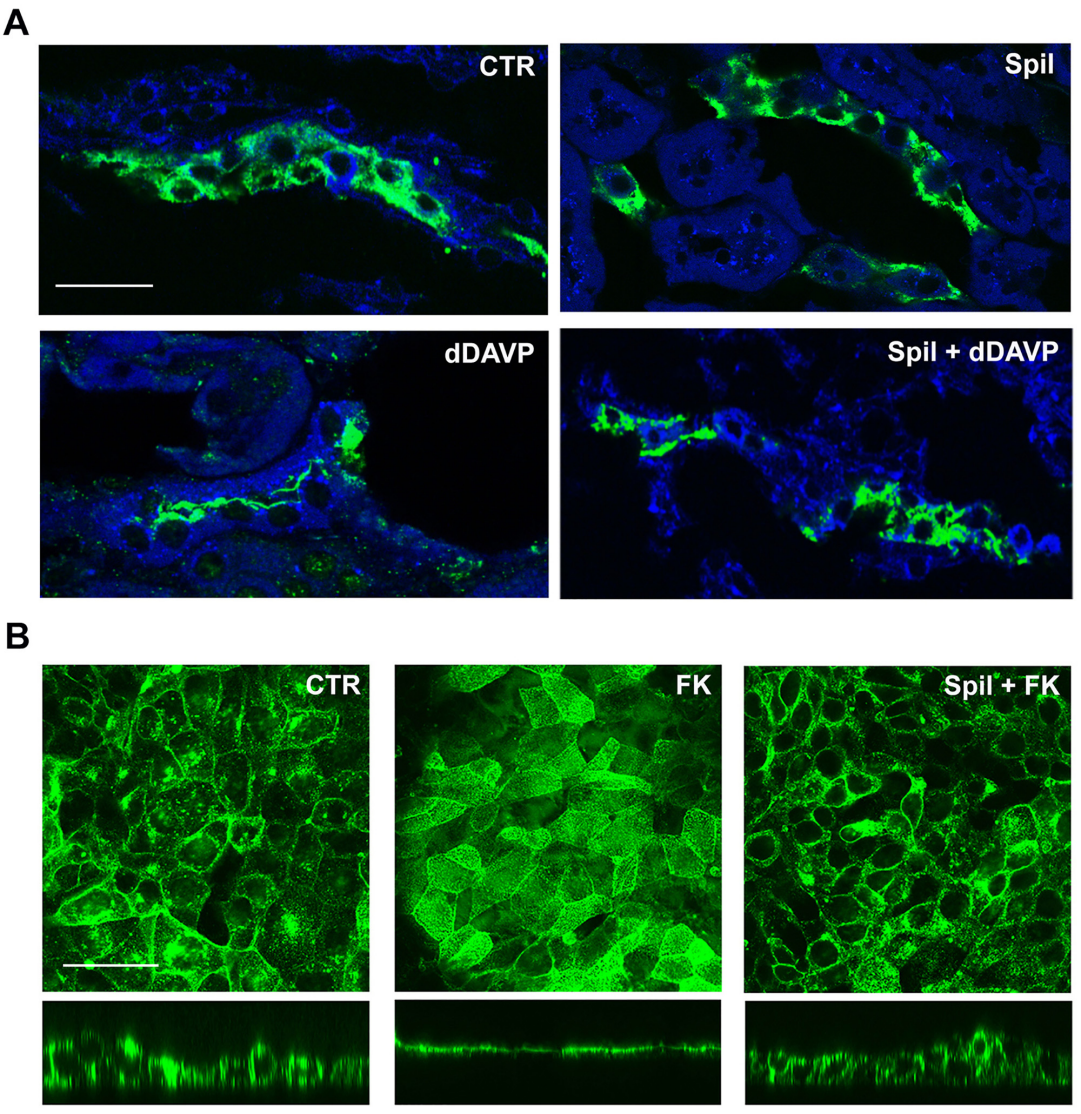
Spilanthol decreases apical plasma membrane expression of AQP2 in freshly isolated kidney slices and MCD4 cells. **(A)** Immunofluorescence analysis of AQP2 subcellular localization in freshly isolated kidney slices in resting condition (Ctr), after 100 μg/ml spilanthol stimulation (Spil), and after incubation with dDAVP in the absence (dDAVP) or in the presence of the spilanthol (Spil + dDAVP). Compared to Ctr and Spil conditions, dDAVP redistributed AQP2 staining to the apical plasma membrane. Of note, spilanthol prevented the dDAVP-induced effect on AQP2 membrane accumulation. Bar = 30 μm. **(B)** Immunofluorescence analysis of AQP2 subcellular localization in MCD4 renal cells in resting condition and after incubation with forskolin (FK) in the absence or in the presence of 100 μg/ml spilanthol (Spil + FK). AQP2 immunostaining was visualized in the xy apical confocal plan (upper panels) and in the xz confocal plan (lower panels). Compared to Ctr conditions, FK redistributed AQP2 staining to the apical plasma membrane. Spilanthol prevented the FK-induced effect on AQP2 membrane accumulation. Bar = 20 μm. Pictures are representative of at least three independent experiments giving the same results.

We then focused our attention on the AVP-dependent AQP2 accumulation at the apical plasma membrane of CD, another important player in the urinary concentrating mechanism. At first, the study was performed on freshly isolated kidney slice. In order to physiologically stimulate AQP2 exposure on the apical membrane of the cells lining the CD we used the AVPR2 specific agonist dDAVP. As shown in Fig 5A, we analyzed AQP2 subcellular localization in freshly isolated kidney slices in resting condition (CTR), after 100 μg/ml spilanthol exposure (Spil), and after stimulation with dDAVP in the absence (dDAVP) or in the presence of spilanthol (Spil+dDAVP). Spilanthol did not increase the rate of AQP2 apical exposure when compared to kidney slices in control condition. Of note, exposure to spilanthol largely prevented the dramatic redistribution of AQP2 on the apical membrane induced by the exposure to dDAVP in freshly isolated kidney slices.

We then assessed the putative inhibitory effect of spilanthol on AQP2 traslocation on MCD4 cells, a clone of M-1 cells stably transfected with human-AQP2 [20–22]. Fig 5B reports the confocal analysis of AQP2 subcellular localization in MCD4 renal cells in unstimulated condition and after incubation with the AC activator forskolin (FK), either in the absence or in the presence of 100 μg/ml spilanthol (Spil+FK). Compared to the prevalent cytosolic AQP2 staining in resting conditions, the increased cAMP level induced by exposure to forskolin clearly redistributed AQP2 staining at the apical plasma membrane of MCD4 cells. Of note, 30 min pre-exposure to 100 μg/ml spilanthol significantly prevented the FK-induced effect on AQP2 membrane accumulation as observed both in a xy confocal plan, passing through the cell apical membrane (upper panel), and in the xz confocal plan.

Altogether, these findings indicate that spilanthol effectiveness as diuretic seems to be related to its inhibitory effects on both NKCC2 phosphorylation in the TAL and AQP2 accumulation at the apical plasma membrane of CD.

We wondered therefore to verify whether spilanthol was able to increase diuresis as well as salt urinary excretion when administered *in vivo*.

### *In vivo* effect of acute administration of spilanthol on urinary parameters

Twenty-four-hour urine samples were obtained by placing the mice in metabolic cages, 6 for each group. As shown in Table 1, 800 mg/Kg *per os*.(p.o.) spilanthol-treated mice exhibited a significant 2-fold increase in urine output compared with control mice (ml/24h: 2.23 ± 0.202 *vs* 1.05 ± 0.180, n = 6 per group, p<0.01). This observation was associated with a markedly reduced urine osmolality compared with control mice (milliosmoles/kg/24h: 1697 ± 309.5 *vs* 3083 ± 196.5, n = 6 per group, p<0.05). In addition, spilanthol-induced diuresis was accompanied by an increase in sodium (mEq/24h: 0.210 ± 0.017 vs. 0.123 ± 0.018, p<0.05), potassium (mEq/24h: 0.200 ± 0.019 vs. 0.130 ± 0.011, p<0.05) and chloride (mEq/24h: 0.31 ± 0.023 *vs* 0.21 ± 0.025, p<0.05) excretion.

**Table 1:** 24-h urine output, urine osmolality and renal electrolyte excretion in control and spilanthol treated mice (800 mg/kg p.o.).

Urine Parameter	Control	Spilanthol	
volume (ml)/24 h	1.05 ± 0.180	2.23 ± 0.202	**
Osmolality (mOsmol/kg)/24 h	3083 ± 196.5	1697 ± 309.5	**
Na^+^ mEq/24 h	0.123 ± 0.018	0.21 ± 0.017	*
K^+^ mEq/24 h	0.13 ± 0.011	0.20 ± 0.019	*
Cl^−^ mEq/24 h	0.21 ± 0.025	0.31 ± 0.023	*

Values are means ± SEM of measurements in n = 6 mice/group. Statistical analysis was performed using Student**’**s T-test for unpaired data.

*p<0,05,

**p<0,01

These results clearly suggested that orally administered spilanthol is able to mimic the effect of furosemide, strongly supporting the conclusion from our *ex vivo* and *in vitro* studies.

We therefore aimed at understanding at cellular level the mechanisms by which spilanthol exerted the effects reported so far on the urine concentrating mechanisms.

### Role of spilanthol in terminating cAMP signaling

**Fig 6:**
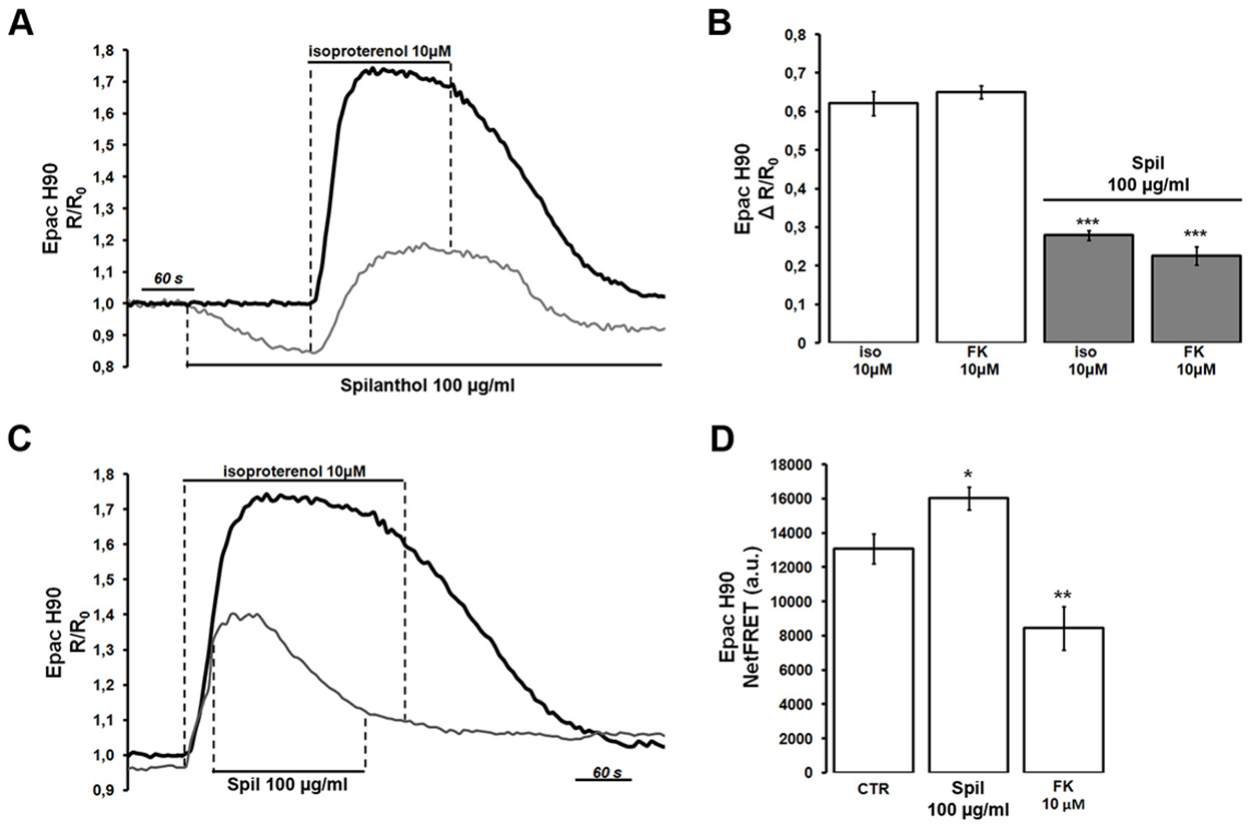
Stimulation of NKCC2-HEK293 cells with spilanthol both prevents and reverses isoproterenol-induced cAMP formation as measured by the 480/535 nm emission ratio of the Epac sensor H90. **(A)** The grey trace shows that the response to a supramaximal dose of 10 μM isoproteranol is reduced by pretreatment with 100 μg/ml spilanthol. Note that exposure to spilanthol by itself reduced basal cAMP levels. The black trace represents control recording not exposed to spilanthol. **(B)** Statistical analysis revealed that spilanthol inhibited the production of cAMP induced by both 10 μM isoproterenol (***p<0.001) and 10 μM forskolin (***p<0.001). Comparable results were obtained in 4 different runs and significance calculated by Student**’**s T-test for unpaired data. **(C)** The grey trace shows that acute addition of 100 μg/ml spilanthol during stimulation with 10 μM isoproteranol reverses the ratio elevation. The black trace represents control recording not exposed to spilanthol. **(D)** H90-transfected NKCC2-HEK293 cells were either left under basal condition (resting) or treated with 100 μg/ml spilanthol (60 min) or 10 μM forskolin (30 min). Compared with untreated cells, spilanthol significantly increased netFRET signal (*p<0.05 *vs* untreated). On the other hand, forskolin significantly reduced netFRET signal when compared with untreated cells (**p<0.01 *vs* untreated). Unpaired data were assessed for statistical significance using the Student’s t test. Data are expressed as means ± SEM with n equal to the total number of cells analyzed.

Because AVPR2 activation enhances NKCC2 phosphorylation and AQP2 membrane accumulation primarily via increases in intracellular cAMP, we assessed whether or not exposure to 100 μg/ml spilanthol might impinge cAMP signaling induced by a physiological agonist such as isoproterenol in NKCC2-HEK293 cells. The representative grey trace in Fig 6A shows that exposure to spilanthol by itself reduced cAMP levels, as measured by the FRET-based probe H90. Under the continuous presence of spilanthol, the large elevation of the emission ratio induced by 10 μM isoproterenol was partly abolished as evident by comparison with the black trace representing a cell not exposed to spilanthol (Fig 6A). In addition, exposure to spilanthol also reduced the elevation of intracellular cAMP level induced by forskolin (Fig 6B, FK), which directly activates AC. Statistical analysis revealed that spilanthol significantly inhibited the production of cAMP induced by both 10 μM isoproterenol (ΔR/R_0_: 0.621 a.u. ± 0.031, n = 28 cells *vs* 0.279 a.u. ± 0.012, n = 25 cells, ***p<0.001) and 10 μM forskolin (ΔR/R_0_: 0.651 a.u.±0.017, n = 28 cells *vs* 0.226 a.u. ± 0.024, n = 25 cells, ***p<0.001), respectively.

Interestingly, spilanthol exposure was also able to reverse cAMP production in cells previously stimulated with isoproterenol. The representative grey trace in Fig 6C indicated that spilanthol addition, during the isoproterenol-induced cAMP elevation, caused a rapid reversal of the emission ratio, as measured with the Epac-based probe. The representative black trace indicates control recording not exposed to spilanthol. These data indicate that short-time exposure (1–5 min) to spilanthol was able to reduce or reverse agonist-induced cAMP production in NKCC2-HEK293 cells.

We then wondered whether or not a longer exposure to spilanthol (60 min) similar to that used to evaluate its effect on NKCC2 phosphorylation was able to affect intracellular cAMP levels. Steady state FRET experiments were performed with Epac-H90, which reports increases in cAMP levels as reduction of NetFRET signal (Fig 6D). H90-transfected NKCC2-HEK293 cells were either left under basal condition (resting) or treated with 100 μg/ml spilanthol (60 min) or 10 μM forskolin (30 min). Compared with untreated cells (NetFRET: 13073.82 a.u. ± 880.18, n = 128 cells), long-term exposure to spilanthol significantly increased netFRETsignal (NetFRET: 16013.22 a.u. ± 661.98, n = 115 cells, *p<0.05 *vs* untreated) indicating a reduction of basal intracellular cAMP. On the other hand, forskolin significantly reduced net FRET signal when compared with untreated cells (NetFRET: 8445.308 a.u. ± 1267.65, n = 120 cells, **p<0.01 *vs* untreated).

Altogether these results indicate that both short- and long-term exposure to spilanthol rapidly reduced or reversed basal and agonist-induced increases of cAMP levels in NKCC2-HEK293 cells, respectively.

**Fig 7:**
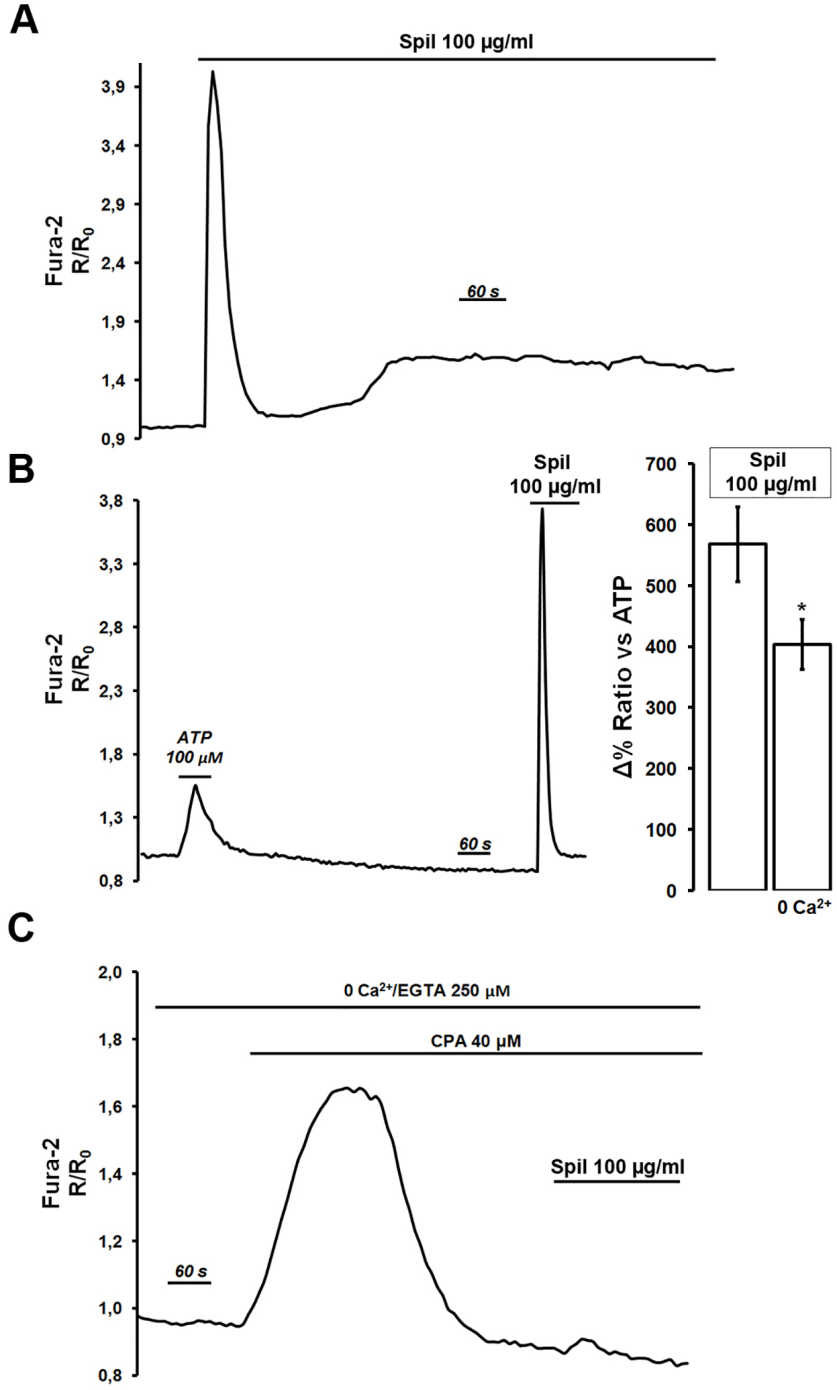
Spilanthol increases intracellular Ca^2+^ as measured in Fura-2-loaded NKCC2-HEK293 cells. **(A)** Exposure to spilanthol (15 min, 100 μg/ml) induced a biphasic increase in intracellular Ca^2+^ level: a fast and transient peak followed by a slow and sustained increase of the basal Ca^2+^ level. Representative trace of n = 82 cells in 4 different experiments. **(B)** Spilanthol-induced intracellular Ca^2+^increase was compared whenever possible with response induced by a maximal dose of the Ca^2+^-mobilizing agonist ATP (100 μM) used in all the experiments performed as internal control. Representative trace of n = 75 cells in 4 different experiments. Right inset shows data indicating that spilanthol-induced Ca^2+^ increase was significantly reduced in the absence of extracellular Ca^2+^ (*P<0.05). Unpaired data were assessed for statistical significance using the Student’s t test. Data are expressed as means ± SEM with n equal to the total number of cells analyzed. **(C)** The intracellular Ca^2+^ increase exerted by exposure to 100 μg/ml spilanthol was completely prevented after CPA/0 Ca^2+^-induced ER-emptying. Representative trace of n = 65 cells in 4 different experiments.

In view of previous studies in which Ca^2+^-mediated agonists were found to reduce intracellular cAMP levels in renal cells [31], parallel experiments were performed with Fura-2 loaded NKCC2-HEK293 cells to examine whether spilanthol had any effect on intracellular Ca^2+^ signaling. Results shown in Fig 7A indicate that in the presence of 1.2 mM extracellular Ca^2+^ in the perfusing solution, 15–20 min exposure to 100 μg/ml spilanthol elicited a biphasic cytosolic Ca^2+^ increase: a fast and large transient followed by a slow increase in intracellular calcium levels. Fig 7B showed that the cytosolic Ca^2+^transient induced by spilanthol was significantly larger than that produced by a sub-maximal dose of the Ca^2+^-mobilizing agonist ATP (100 μM) used in the same cells as internal control. In addition, the data in Fig 7B right inset, showed that spilanthol-induced Ca^2+^ increase (Δ% Ratio *vs* ATP: 568.44% ± 61.24, n = 75 cells) was significantly reduced in the absence of extracellular Ca^2+^ (Δ% Ratio *vs* ATP: 403.89% ± 41.40, n = 68 cells, *P<0.05), suggesting that an important component of the Ca^2+^ signal elicited by spilanthol was represented by Ca^2+^ entry across the plasma membrane.

To evaluate the role of internal Ca^2+^ stores in the effect exerted by spilanthol, NKCC2-HEK293 cells were exposed to the sarco/endoplasmic reticulum Ca^2+^-adenosine triphosphatase (SERCA) pump inhibitor cyclopiazonic acid (CPA; 20 μM, 20 min) in the absence of extracellular Ca^2+^ (Fig 7C). CPA-induced depletion of internal Ca^2+^ stores as a result of Ca^2+^ leak from the ER transiently elevates intracellular Ca^2+^ levels. Under these experimental conditions exposure to 100 μg/ml spilanthol did not cause any change in the Fura-2 ratio clearly indicating that spilanthol enhanced both Ca^2+^ influx at the plasma membrane and Ca^2+^ release from the intracellular calcium stores. Similar results were obtained in parallel experiments performed on Fura-2 loaded MCD4 cells (data not shown).

**Fig 8:**
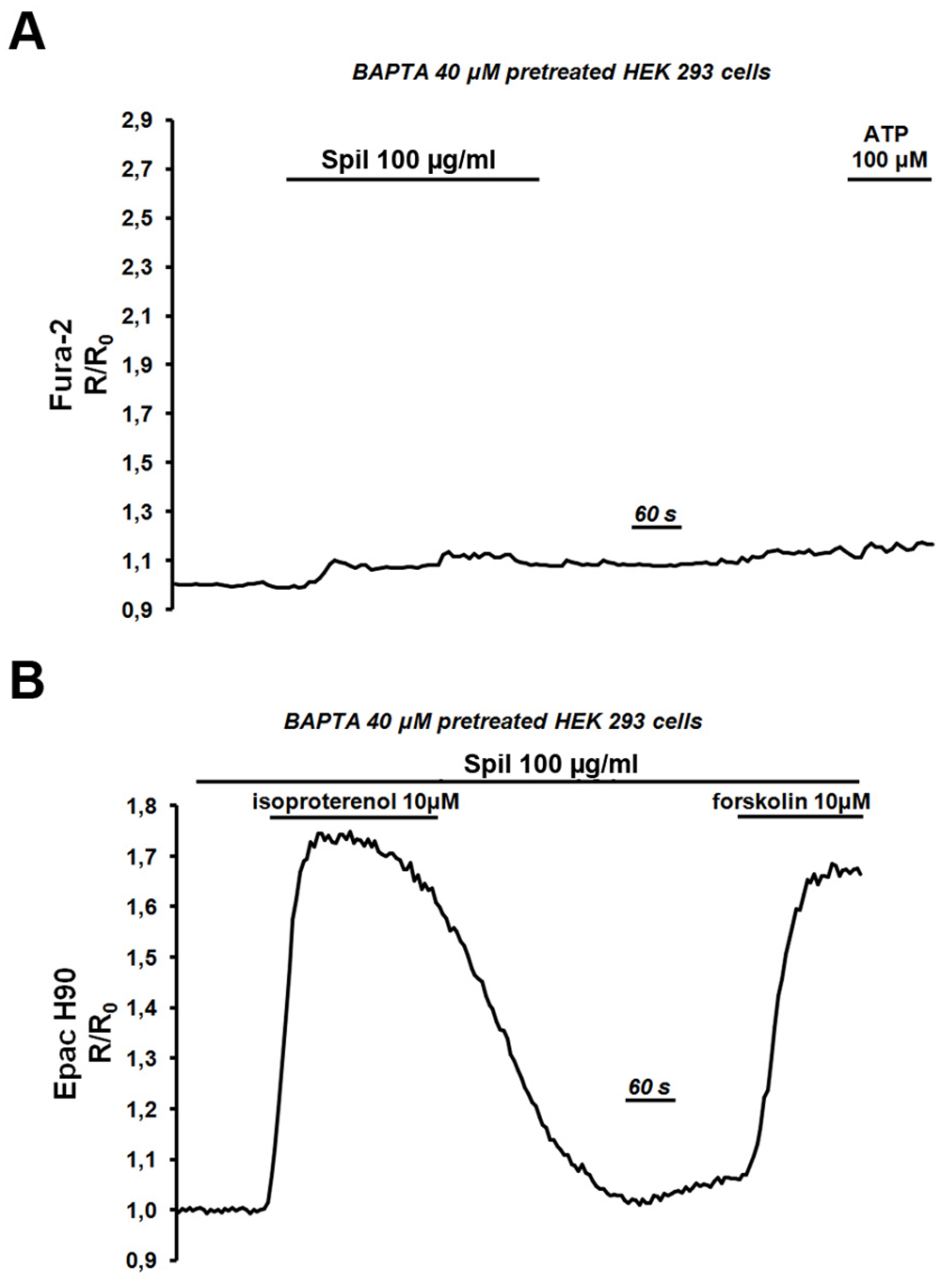
Spilanthol-induced inhibition of cAMP production is sensitive to [Ca^2+^]_i_. **(A)** After pretreatment with BAPTA-AM (30 min, 40 μM) neither exposure to spilanthol 100 μg/ml nor to ATP (100 μM) were able to increase intracellular Ca^2+^ level as measured in Fura-2 loaded NKCC2-HEK293 cells. Representative trace of n = 72 cells in 4 different experiments. **(B)** Under the same experimental condition (BAPTA-AM, 30 min, 40 μM) spilanthol did not inhibit cAMP production induced by either 10 μM isoproterenol or 10 μM forskolin as measured by the 480/535 nm emission ratio of the cAMP/FRET probe. Representative trace of n = 23 cells in 4 different experiments.

We next attempted to evaluate the relative contributions of Ca^2+^ signaling on spilanthol-mediated inhibition of cAMP production in NKCC2-HEK293 cells. During these experiments we eliminated the Ca^2+^ signaling component by pretreating the cells with BAPTA-AM, a cell-permeant highly selective Ca^2+^ chelator. Control experiments (Fig 8A) demonstrated that spilanthol and ATP were not able to induce any increase in intracellular Ca^2+^ levels in BAPTA-pretreated NKCC2-HEK293 cells loaded with Fura-2. Under these experimental conditions the elevation of the Epac H90 emission ratio induced by neither 10 μM isoproterenol (ΔR/R_0_: 0.675 a.u.±0.013) nor 10 μM forskolin (ΔR/R_0_: 0.627 a.u.±0.031) were reduced by pre-exposure to spilanthol (Fig 8B). In addition, spilanthol by itself did not reduce basal cAMP levels, as measured by FRET, clearly indicating that spilanthol exerts its inhibitory action on cAMP signaling working through intracellular Ca^2+^ increases.

## Discussion

Diuretics are one of the most commonly prescribed drugs, used to reduce the abnormal accumulation of excess fluid in the body. They are widely used for the clinical management of hypertension and edema and act by eliminating the excess fluid through increasing the volume of urine excretion reducing Na^+^ and water reabsorption [32]. Considering the growing concern that has been raised about the adverse effects of classic diuretics such as thiazides and loop diuretics [2], new equally effective drugs are interesting and needed.

Extracts from *Acmella oleracea* have been reported to exert potent diuretic effects in rats [13, 33]. Specifically, Ratnasooriya et al demonstrated that the strong diuretic effect evoked by *Acmella oleracea* extract after 1 h was similar to that evoked by the NKCC2 blocker furosemide [13].

Based on these findings we dissected the molecular mechanisms underlying this effect in both freshly isolated mouse kidney slices and cell line models expressing either NKCC2 or AQP2, the main transporters involved in the urinary concentrating process.

NKCC2 phosphorylation at the 3 N-terminal regulatory threonines is well known to be a) induced by vasopressin [15], b) a prerequisite for NKCC2 activation and transport activity [34], inhibited by furosemide [34].

Interestingly, we found that the main constituent of *Acmella oleracea*, spilanthol, is able to strongly inhibit NKCC2 phosphorylation/activation in both resting and activating conditions in freshly isolated kidney slices. Of note, when orally administered to mice, spilanthol exerts a significant diuretic effect concomitantly to increased kaliuretic and natriuretic responses suggesting that it is acting as a furosemide-like diuretic.

Of note, we also found that spilanthol inhibits vasopressin-induced AQP2 translocation in mouse CD cells, which is a prerequisite for water reabsorption during anti-diuresis in physiological conditions suggesting that spilanthol might act also as ‘acquaretic’.

The reduced efficiency in the urine concentrating mechanisms reflects, at cellular level, a complex interplay between Ca^2+^ and cAMP elicited by exposure to spilanthol. We demonstrated for the first time that spilanthol significantly increased intracellular Ca^2+^ levels in renal cells with a mechanism that involves both Ca^2+^ influx at the plasma membrane and Ca^2+^ release from the ER. Nonetheless, whether spilanthol is activating a Gαq-protein coupled receptor or it is acting like a Ca^2+^-specific ionophore, is still without an answer. Interestingly, spilanthol is responsible for the tingling sensate induced by *Acmella oleracea*. It is indeed likely that, like other alkamides inducing same sensate such as capsaicin, spilanthol may activate TRPA1, a specific transient receptor potential (TRP) [35].

Importantly, we found that the spilantol-induced cytosolic Ca^2+^ increase significantly reduces cAMP levels in renal cells. It is well recognized that both NKCC2 activation and AQP2 membrane expression are cAMP-mediated mechanisms. NKCC2 has been demonstrated to be phosphorylated and shuttled into the apical membrane of the TAL in response to vasopressin [15] and cAMP elevation [36] in a PKA-dependent manner [37]. The cAMP-mediated AQP2 phosphorylation and translocation toward the apical membrane of CD has been also extensively demonstrated [17].

In many cell types, the intracellular Ca^2+^ concentration regulates cAMP levels through interactions of Ca^2+^ on cAMP synthesis and/or cAMP hydrolysis. These effects of [Ca^2+^]_i_ are linked to the presence of Ca^2+^-sensitive adenylyl cyclases (ACs) and/or Ca^2+^/calmodulin-dependent phosphodiesterases (PDEs). Importantly, high Ca^2+^ suppresses cAMP production in response to forskolin inhibiting the AVP-dependent AC in medullary TAL (mTAL) in rat kidneys [38]. Moreover, de Jesus Ferreira et al. described coexpression of Ca^2+^ inhibitable AC type 6 (AC6) and the Calcium Sensing Receptors (CaSR) in the cortical TAL of the kidney and further showed potent antagonism of cAMP signaling during CaSR stimulation by physiological levels of extracellular [Ca^2+^][31]. In addition, AC6 was found to be functionally expressed in CD and involved in the regulation of cAMP production in at this site [39]. We performed functional experiments in HEK293 cells, which endogenously express many isoforms of AC (AC1, 3, 5, 6, 7, and 9 and soluble, bicarbonate-sensitive AC; [40–42], of which some are inhibitable by Ca^2+^ (e.g., AC5 and AC6), and which do not express Ca^2+^/calmodulin-dependent phosphodiesterases [43]. Indeed, our functional studies suggest that *in vivo* spilanthol, inducing a massive increase in intracellular [Ca^2+^] may act inhibiting the AC6 rather than activating Ca^2+^/calmodulin-dependent PDEs in TAL and CD.

However, it has also been reported that both NKCC2 and AQP2 might be regulated by PDEs. For instance, PDE type 4 (PDE4) blunts the stimulatory effect of β-adrenergic receptor stimulation on NKCC2 trafficking in TALs suspension from rat kidneys [44]. PDE4 also inhibits AQP2 shuttling at the plasma membrane [45] and its hyperactivity causes nephrogenic diabetes insipidus (NDI) in a mouse model [38], thus suggesting a crucial role of PDEs in the diuresis.

Among the PDE members family PDE1 was reported to be efficiently activated by the Ca^2+^/calmodulin complex [46] and expressed in both TAL and CD [47]. Therefore, we cannot exclude that spilanthol *in vivo* may activate the Ca^2+^/calmodulin-sensitive PDE1 in the kidney tubules.

## Conclusions

Collectively these data are of extreme pathophysiological importance since they indicate that spilanthol is able to mimic the diuretic effect of furosemide *in vivo* targeting intracellular pathways in the kidney upstream to both NKCC2 and AQP2 thus suggesting spilanthol as a novel powerful molecule in the pharmacological field of diuretics.

Considering the growing concern that has been raised about the adverse effects of classic diuretics such as thiazides and loop diuretics, new equally effective synthetic, semi-synthetic or natural sources (herbs and botanicals) drugs are interesting and needed.

Furthermore, applying to spilanthol the technologies to specifically deliver therapeutic molecules in the kidney (for review see [48]) would minimize the off-target effects and enhance its renal efficacy within the renal tissue.

## Supporting Information

S1 Fig Western blot analysis using T4 and R5 antibody on both untransfected and NKCC2 expressing HEK293 cells. A) Expression of NKCC cotransporters in lysates from untransfected (HEK293) and NKCC2-expressing HEK293 cells (HEK-NKCC2), using T4 antibody recognizing both NKCC1 and NKCC2 (Developmental Studies Hybridoma Bank, University of Iowa, Department of Biology, Iowa City, Iowa; http://dshb.biology.uiowa.edu/Na-K-Cl-). The expression of endogenous NKCC1 in HEK293 cells was barely detectable compared to the expression of NKCC2 in NKCC2-transfected HEK293 cells, used in the *in vitro* study. B) Western Blotting using either T4 antibody (WB T4 antibody) or R5 antibody (WB R5 antibody) on NKCC1/2 immunoprecipitates from untransfected (HEK293) and NKCC2-expressing HEK293 cells (HEK-NKCC2) lysates. T4 antibody, used to immunoprecipiate both NKCC1 and 2, was unable to immunoprecipitate the endogenously expressed NKCC1 in HEK293 cells, most likely for the low level of NKCC1 expression in HEK293 cells. Thus, R5 antibody was able to recognize only NKCC2 immunoprecipitated from NKCC2-expressing HEK293 cells lysate (WR R5 antibody, pNKCC1/2). C) Western blotting using R5 antibody on lysates from untransfected (HEK293) and NKCC2-expressing HEK293 cell (HEK-NKCC2) either in resting (CTR) or activating conditions (low Cl^−^). R5 antibody showed a faint signal at the molecular weight corresponding to NKCC, in untransfected HEK293 cells, which however did not increase in low Cl^−^ activating conditions.
(JPG)

S2 Fig Effect of increasing dose of spilanthol on NKCC2 phosphorylation in renal cells. **Left panel.** NKCC2-HEK293 cells were stimulated overnight with the indicated amount of spilanthol (10, 50, 100 μg/ml) then lysed and total protein extracts analyzed for pNKCC2 expression as shown by this representative Western blot. Right panel. Densitometric analysis showed a significant reduction of pNKCC2 (normalized to total NKCC2) in NKCC2-HEK293 cells proportional at the concentration of spilanthol used (***p<0.001, **p<0.01) compared to control condition. Comparable results were obtained in three different experiments and significance calculated by Student’s T-test for unpaired data.
(JPG)
