# Supplementary figures and images for: Spilanthol from Acmella Oleracea Lowers the Intracellular Levels of cAMP Impairing NKCC2 Phosphorylation and Water Channel AQP2 Membrane Expression in Mouse Kidney
Source: PLoS One. 2016 May 23;11(5):e0156021. doi: 10.1371/journal.pone.0156021 (PMC4877099; doi:10.1371/journal.pone.0156021)

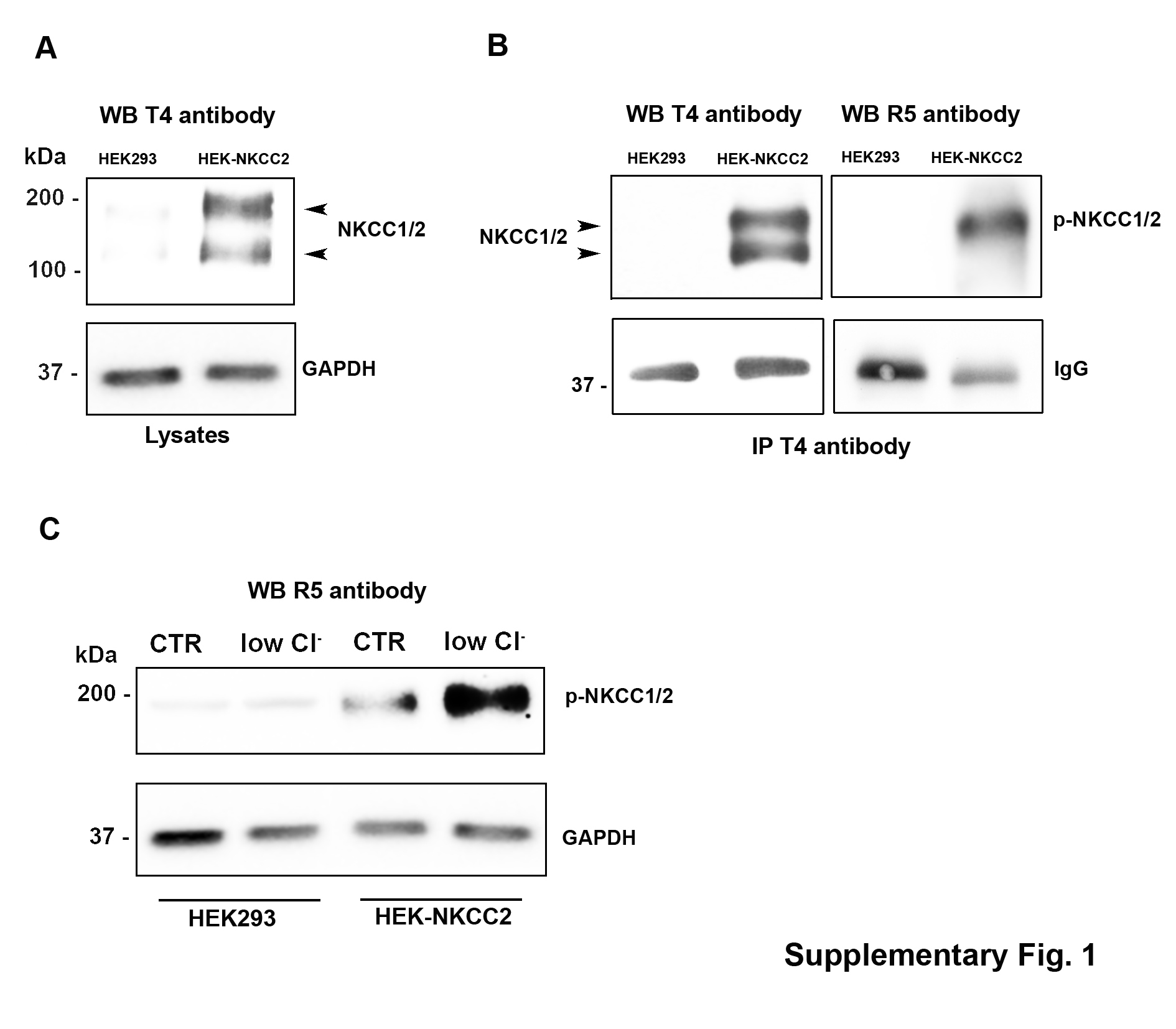

Supplement: S1 Fig — A) Expression of NKCC cotransporters in lysates from untransfected (HEK293) and NKCC2-expressing HEK293 cells (HEK-NKCC2), using T4 antibody recognizing both NKCC1 and NKCC2 (Developmental Studies Hybridoma Bank, University of Iowa, Department of Biology, Iowa City, Iowa; http://dshb.biology.uiowa.edu/Na-K-Cl-). The expression of endogenous NKCC1 in HEK293 cells was barely detectable compared to the expression of NKCC2 in NKCC2-transfected HEK293 cells, used in the in vitro study. B) Western Blotting using either T4 antibody (WB T4 antibody) or R5 antibody (WB R5 antibody) on NKCC1/2 immunoprecipitates from untransfected (HEK293) and NKCC2-expressing HEK293 cells (HEK-NKCC2) lysates. T4 antibody, used to immunoprecipiate both NKCC1 and 2, was unable to immunoprecipitate the endogenously expressed NKCC1 in HEK293 cells, most likely for the low level of NKCC1 expression in HEK293 cells. Thus, R5 antibody was able to recognize only NKCC2 immunoprecipitated from NKCC2-expressing HEK293 cells lysate (WR R5 antibody, pNKCC1/2). C) Western blotting using R5 antibody on lysates from untransfected (HEK293) and NKCC2-expressing HEK293 cell (HEK-NKCC2) either in resting (CTR) or activating conditions (low Cl−). R5 antibody showed a faint signal at the molecular weight corresponding to NKCC, in untransfected HEK293 cells, which however did not increase in low Cl− activating conditions. (JPG) [file pone.0156021.s001.jpg]

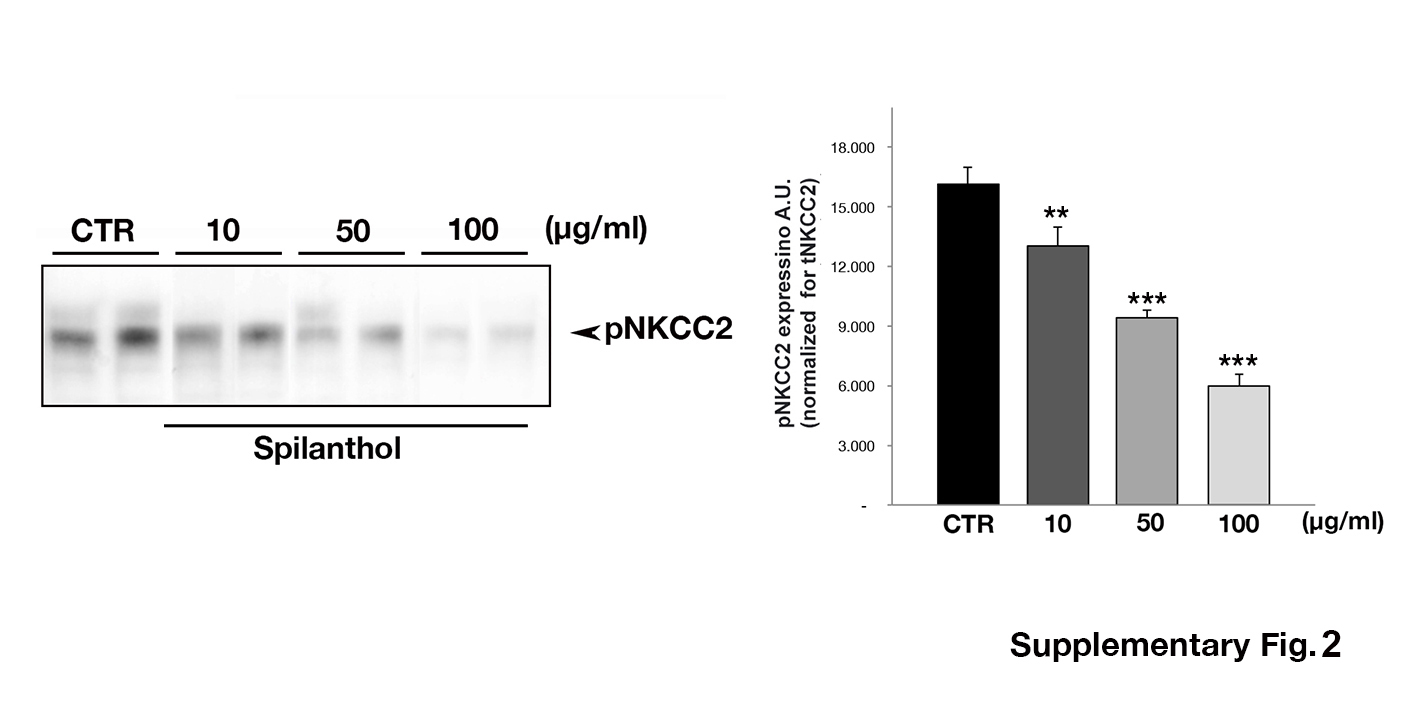

Supplement: S2 Fig — Left panel. NKCC2-HEK293 cells were stimulated overnight with the indicated amount of spilanthol (10, 50, 100 μg/ml) then lysed and total protein extracts analyzed for pNKCC2 expression as shown by this representative Western blot. Right panel. Densitometric analysis showed a significant reduction of pNKCC2 (normalized to total NKCC2) in NKCC2-HEK293 cells proportional at the concentration of spilanthol used (***p<0.001, **p<0.01) compared to control condition. Comparable results were obtained in three different experiments and significance calculated by Student’s T-test for unpaired data. (JPG) [file pone.0156021.s002.jpg]
